# Word Sense Disambiguation with Wikipedia Entities: A Survey of Entity Linking Approaches

**DOI:** 10.3390/e28020236

**Published:** 2026-02-18

**Authors:** Michael Angelos Simos, Christos Makris

**Affiliations:** Department of Computer Engineering and Informatics, University of Patras, 26504 Patras, Greece

**Keywords:** word sense disambiguation, entity linking, Wikipedia, knowledge bases, semantic relatedness, sense embeddings, transformers, multilingual NLP

## Abstract

The inference of unstructured text semantics is a crucial preprocessing task for NLP and AI applications. Word sense disambiguation and entity linking tasks resolve ambiguous terms within unstructured text corpora to senses from a predefined knowledge source. Wikipedia has been one of the most popular sources due to its completeness, high link density, and multi-language support. In the context of chatbot-mediated consumption of information in recent years through implicit disambiguation and semantic representations in LLMs, Wikipedia remains an invaluable source and reference point. This survey covers methodologies for entity linking with Wikipedia, including early systems based on hyperlink statistics and semantic relatedness, methods using graph inference problem formalizations and graph label propagation algorithms, neural and contextual methods based on sense embeddings and transformers, and multimodal, cross-lingual, and cross-domain settings. Moreover, we cover semantic annotation workflows that facilitate the scaled-up use of Wikipedia-centric entity linking. We also provide an overview of the available datasets and evaluation measures. We discuss challenges such as partial coverage, NIL concepts, the level of sense definition, combining WSD and large-scale language models, as well as the complementary use of Wikidata.

## 1. Introduction

Lexical ambiguity is pervasive in natural language; many words, multiword expressions, and named entities can be interpreted in multiple ways depending on context. *Word sense disambiguation* (WSD) is the task of assigning the appropriate sense label to each fragment in a text, given a predefined sense inventory. In parallel, *entity linking* (EL), also called named entity disambiguation, maps mentions of entities such as persons, organizations, or locations to entries in a knowledge base, commonly Wikipedia.

Traditionally, WSD has been studied with lexicographic resources such as WordNet, whereas EL has been studied with encyclopedic resources such as Wikipedia and DBpedia. The division between these communities is increasingly porous. Many systems treat both common nouns and named entities uniformly as *concepts* or *entities* in a large semantic network. Wikipedia plays a crucial role in this development: it offers millions of pages that can act as fine-grained sense identifiers for both common and named entities, connected by hyperlinks, redirects, categories, and infoboxes.

Importantly, in many contemporary information access settings, Wikipedia is no longer consumed primarily through direct page visits. Users increasingly interact with Wikipedia-mediated knowledge through search engines, voice assistants, and chatbot interfaces powered by large language models (LLMs). In this ecosystem, Wikipedia often acts as an underlying high-quality training input; it provides stable page-level anchors, textual descriptions, and link structure that support retrieval, grounding, and evaluation.

In this paper, we adopt the perspective that *word sense disambiguation with Wikipedia entities* is a form of *word sense linking*, where the target inventory consists of Wikipedia pages (often enriched or unified with other resources such as WordNet). This perspective encompasses named entity linking, concept linking, and unified WSD/EL models [1,2]. It emphasizes that resolving ambiguity in text increasingly means grounding language in large, evolving knowledge bases rather than in small, fixed lexicons.

### 1.1. Illustrative Example

To make the unification between common-noun WSD and named-entity linking concrete, Table 1 shows two short cases. In both, the model must (1) generate a small set of plausible Wikipedia pages and (2) select the best page given the surrounding context, optionally returning an NIL decision when no suitable page exists.

### 1.2. Motivation

Wikipedia has several properties that make it attractive as a sense inventory for WSD and EL:

- **Coverage:** Wikipedia covers an extremely large number of entities and concepts, including named entities, domain-specific concepts, and many terms not present in traditional lexicons.
- **Structure:** Hyperlinks, redirects, disambiguation pages, categories, and infoboxes provide rich structural information that can be exploited for semantic relatedness and graph-based reasoning.
- **Textual context:** Each page contains descriptive text whose content can be used to learn semantic representations and to compare candidate senses with contextual words.
- **Multilinguality:** Inter-language links and multilingual editions support cross-lingual disambiguation and sense alignment.

These properties enable approaches that extend beyond assigning discrete sense identifiers; instead, systems can leverage a combination of graph structure, distributional semantics, and multilingual signals.

At the same time, Wikipedia-based WSD poses challenges. Wikipedia is dynamic; pages are added, deleted, split, or merged. The granularity of Wikipedia pages does not always align with traditional sense inventories, and coverage varies across domains and languages. Furthermore, modern neural models encode substantial implicit world knowledge that must be reconciled with explicit Wikipedia-based knowledge.

### 1.3. Scope and Focus

Existing surveys on WSD [2,3] provide comprehensive overviews of algorithms and resources, but they treat Wikipedia-based methods as one component among many. In contrast, the present survey focuses specifically on methods where the following apply:

- Wikipedia or a resource directly derived from it (e.g., BabelNet) is the primary sense inventory or a key part of a unified inventory;
- The task is to disambiguate words or mentions in context by linking them to Wikipedia pages or Wikipedia-based sense identifiers;
- The systems explicitly exploit Wikipedia’s structure (anchors, links, categories, redirects) or textual content for modeling.

Within this scope, we cover the following:

1. Early Wikipedia-based WSD approaches that exploit hyperlink statistics and semantic relatedness [4,5,6,7,8];
2. Unified WSD and EL frameworks such as Babelfy [1] and graph-based label propagation methods over Wikipedia-WordNet networks;
3. Supervised and semi-supervised methods that automatically construct sense-annotated corpora from Wikipedia, such as Train-O-Matic [9] and MuLaN [10];
4. Neural and contextualized methods, including sense embeddings and transformer-based WSD [11,12,13,14,15];
5. Multilingual, multimodal, and low-resource settings [10,16,17];
6. Wikipedia-based semantic annotation pipelines and named entity disambiguation frameworks [18,19,20,21];
7. Domain-specific applications and broader word sense linking beyond traditional benchmarks [22,23,24].

### 1.4. Research Questions

We structured this survey around five research questions (RQs) that recur across this paper:

- **RQ1 (Inventory design):** How is Wikipedia operationalized as a sense or entity inventory (pages, redirects, disambiguation pages, and derived resources such as BabelNet), and what design choices affect granularity and coverage?
- **RQ2 (Pipelines and inference):** How do Wikipedia-grounded systems decompose the problem into candidate generation, local scoring, and global inference, and when is collective (graph-based) reasoning beneficial?
- **RQ3 (Representation learning):** How do neural and contextualized models encode mention and page semantics, and how are these representations combined with explicit Wikipedia structure?
- **RQ4 (Multilinguality and robustness):** What methods extend Wikipedia-grounded disambiguation across languages, modalities, and low-resource settings, and what failure modes arise from uneven Wikipedia coverage?
- **RQ5 (LLM era):** How does Wikipedia-grounded WSD and EL relate to implicit disambiguation in LLMs, and why does explicit grounding remain methodologically important (e.g., for auditability and stable evaluation)?

We explicitly revisit and answer these RQs in Section 6.6.

### 1.5. Contributions

The contributions of this survey are as follows:

- We introduce a taxonomy of Wikipedia-based WSD and EL approaches, highlighting common design dimensions and linking them to representative methods.
- We provide a detailed review of methods that use Wikipedia entities as sense identifiers, from early relatedness-based models to modern neural architectures.
- We describe multilingual and multimodal extensions in which Wikipedia acts as a pivot knowledge base and discuss large-scale semantic annotation pipelines grounded in Wikipedia.
- We discuss evaluation benchmarks and metrics for Wikipedia-based WSD and EL and analyze methodological challenges related to sense granularity, NIL entities, and dynamic inventories.
- We identify open research questions and future directions, including integration with large language models and alignment with structured knowledge bases such as Wikidata.

### 1.6. Organization

The remainder of this paper is organized as follows. Section 3 introduces the core definitions for WSD, EL, and Wikipedia-derived semantic resources and presents the taxonomy used throughout the survey. Section 4 reviews algorithmic approaches, spanning early Wikipedia-based WSD, unified WSD/EL formulations over Wikipedia and BabelNet, neural and contextualized models, and multilingual, multimodal, and low-resource extensions. Section 5 summarizes the evaluation practice (datasets, benchmarks, and metrics). Section 6 discusses open challenges and future directions, including the interface between explicit Wikipedia grounding and LLM-based systems. Section 7 concludes the paper.

## 2. Materials and Methods

This survey followed a reproducible literature review protocol designed to (1) retrieve candidate publications about Wikipedia-grounded WSD and EL, (2) screen them with explicit inclusion and exclusion criteria, and (3) extract a standardized set of attributes that support a consistent taxonomy and synthesis.

### 2.1. Protocol Overview

**Figure 1:**
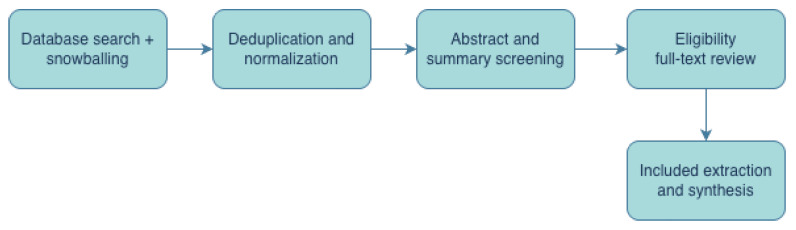
Survey protocol used for study identification, screening, extraction, and synthesis. The protocol is defined to be re-runnable; in a reproducible survey, the final query timestamp and data extraction sheet should be archived with the manuscript.

Figure 1 summarizes the workflow used to identify relevant studies and to standardize extraction and synthesis.

### 2.2. Information Sources and Search Strategy

We queried major digital libraries and indexing services commonly used in NLP, IR, and AI (e.g., ACL Anthology, ACM Digital Library, IEEE Xplore, SpringerLink, and arXiv). We additionally performed backward and forward snowballing from frequently cited surveys and representative systems and merged near-duplicate records (e.g., arXiv preprints vs. camera-ready versions).

Table 2 gives a query template that can be instantiated per library (with field restrictions such as title: or abstract: where supported). The template is intentionally broad; it targets both traditional WSD terminology and the entity linking or named entity disambiguation literature.

### 2.3. Eligibility Criteria

A publication was included if it satisfied all of the following:

- **Wikipedia grounding:** Wikipedia (or a directly derived resource such as BabelNet) is used as the primary inventory for disambiguation or as the target identifier space for linking.
- **Technical specificity:** The paper describes the candidate generation and disambiguation mechanism with sufficient detail to characterize the method (e.g., features or objectives, model architecture, or inference strategy).
- **Empirical support:** The paper reports an evaluation, a benchmark comparison, or a concrete downstream use case with measurable outcomes.

We excluded papers that (1) mention Wikipedia only as background text without explicit grounding, (2) lack methodological detail (e.g., position papers without a described approach), or (3) are non-scholarly artifacts.

### 2.4. Screening Procedure

Screening was performed in two stages. First, titles and abstracts were reviewed to remove clearly irrelevant records and duplicates. Second, the remaining papers were read in full to confirm eligibility and to populate the extraction schema. When multiple versions of the same work existed, we preferred the archival- or camera-ready version.

### 2.5. Extraction Schema

For each included paper, we extracted a standardized set of attributes that enabled reproducible comparison across paradigms:

- **Task framing:** WSD, EL, joint or unified, or word sense linking.
- **Inventory:** Wikipedia pages vs. derived inventories (BabelNet or Wikidata alignment), handling of redirects or disambiguation pages, and NIL policy.
- **Candidate generation:** Anchor statistics, lexical retrieval, dense retrieval, or hybrid.
- **Scoring and inference:** Local context–page matching, graph-based collective inference, neural ranking, or hybrid objectives.
- **Supervision:** Distant supervision from Wikipedia hyperlinks, manually curated gold sets, self-training, or fully unsupervised.
- **Evaluation:** Datasets, metrics, and whether granularity and cross-lingual coverage are analyzed.

### 2.6. Synthesis and Taxonomy Mapping

**Figure 2:**
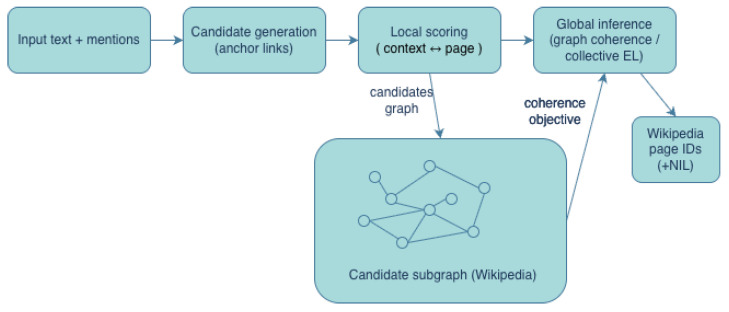
Canonical Wikipedia-grounded WSD/EL pipeline. Systems differ in how candidates are generated (anchors, lexical retrieval, or dense retrieval), how candidates are scored (features or neural matching), and whether global inference is performed (collective or graph-based coherence).

We synthesized the results by mapping each paper onto the taxonomy in Section 3 and by explicitly relating it to the pipeline decomposition in Figure 2. This let us compare systems that shared candidate generation but differed in inference (or vice versa) and separate improvements due to better representations from those due to stronger global coherence objectives.

### 2.7. Transparency Regarding AI-Assisted Tooling

We used limited generative AI assistance for mechanical tasks (e.g., drafting extraction checklists, normalizing terminology, and producing first-pass summaries into the extraction schema). All extracted claims and all synthesized statements were verified manually against the original papers, and the final taxonomy and narrative were authored and validated by the authors.

## 3. Background and Taxonomy

### 3.1. Word Sense Disambiguation

In classical WSD, a sense inventory such as WordNet defines a set of senses for each lemma. Given a sentence, the system must assign to each text fragment word the appropriate sense label. Knowledge-based methods use lexical relations and glosses; supervised methods rely on sense-annotated corpora; unsupervised methods induce senses from usage data; and neural methods employ vector representations and pretrained language models [2,3].

Formally, let $D=(w_{1},\ldots ,w_{n})$ be a document and *T* be the set of target tokens to be disambiguated. For each t∈T, there is a set of candidate senses $S_{t}$ from the inventory. The goal is to select a labeling $y={\left\{y_{t}\right\}}_{t\in T}$ with $y_{t}\in S_{t}$ that maximizes an objective such as P(y∣D) or a scoring function F(D,y), possibly under structural constraints.

### 3.2. Entity Linking

Entity linking (EL) maps mentions in text to entries in a knowledge base (KB), such as Wikipedia. The input is a document *D* and a set of mentions *M*, usually produced by named entity recognition or mention detection. For each mention m∈M, a candidate generator produces a set C(m) of KB entities. A ranking model then selects the most appropriate entity ${\hat{e}}_{m}$ from C(m). Many systems also perform global inference to enforce coherence among the entities selected for different mentions.

The standard pipeline can be summarized as follows:

1. Mention detection: Identify possible mention spans.
2. Candidate generation: Retrieve candidate entities from the KB using lexical and alias information (e.g., anchor texts or redirects).
3. Local scoring: Compute compatibility scores between each candidate and its context.
4. Global inference: Jointly select candidates across the document using graph-based optimization or probabilistic models.

When the KB is Wikipedia, entities correspond to Wikipedia pages, and the system can use anchor statistics, category information, and link structures.

### 3.3. Wikipedia as a Semantic Resource

Wikipedia can be exploited in several ways:

- **Sense inventory:** Each Wikipedia page is treated as a sense or entity; disambiguation amounts to choosing a page.
- **Graph:** Pages, redirects, categories, and hyperlinks form a large graph. Semantic relatedness can be computed by analyzing adjacency and link overlap [8].
- **Textual resource:** Page content provides definitions and example contexts. Methods such as ESA treat pages as dimensions in a concept space [4].
- **Multilingual hub:** Inter-language links connect pages across languages, enabling alignment and transfer.

Wikipedia also underpins derived resources such as BabelNet, which integrates WordNet synsets with Wikipedia pages and other knowledge sources into a single multilingual semantic network [1]. Many Wikipedia-based WSD systems operate on such unified inventories.

### 3.4. Problem Formulation

In the setting considered here, we assumed a KB *K* whose entities correspond to Wikipedia pages or Wikipedia-based synsets (e.g., in BabelNet). Given a document *D* and a set of mentions or target words *M*, the goal is to assign to each m∈M an entity $e_{m}\in K$ or a special NIL label if no appropriate entity exists.

Most systems decompose this problem into candidate generation and candidate selection. Candidate generation often exploits string matching, alias tables built from anchor texts, or search indices over page titles and redirects. Candidate selection uses features derived from the context, the entity content, and the graph structure of *K* or relies on neural architectures that encode these factors implicitly.

### 3.5. Taxonomy of Approaches

Wikipedia-based WSD and EL methods can be according to under several dimensions:

- **Knowledge-based vs. data-driven:** Knowledge-based methods rely primarily on the structure and content of Wikipedia and related graphs, whereas data-driven methods train on labeled instances derived from Wikipedia or external corpora.
- **Local vs. global inference:** Local methods disambiguate each mention independently given its context; global methods jointly disambiguate all mentions in a document or corpus, exploiting coherence.
- **Static vs. contextual representations:** Earlier methods use static vector or graph representations; recent methods employ contextualized representations from transformers.
- **Monolingual vs. multilingual:** Some methods operate on a single language; others exploit cross-lingual signals via inter-language links or multilingual language models.
- **Batch vs. online or distributed:** Certain frameworks are designed for offline processing of corpora; others implement distributed or streaming architectures [19,20].

Table 3 summarizes a high-level taxonomy and lists representative works.

In the following sections, we use this taxonomy to organize the literature.

## 4. Approaches to Wikipedia-Grounded Disambiguation

Wikipedia-grounded WSD and EL systems can be viewed through a shared decomposition into (1) *candidate generation* (producing a small set of plausible Wikipedia pages for each mention), (2) *local scoring* (matching the mentioned context to candidate pages), and (3) *global inference* (enforcing topical coherence across multiple mentions, often via the Wikipedia link graph). Figure 2 provides a canonical schematic that we used to relate methods across paradigms.

### 4.1. Early Wikipedia-Based WSD and Semantic Relatedness

#### 4.1.1. Using Wikipedia for Automatic WSD

Mihalcea [7] was among the first to systematically use Wikipedia for WSD. The central observation is that Wikipedia pages can act as sense descriptions, analogous to dictionary glosses. For a target word, its possible senses correspond to Wikipedia pages reachable from disambiguation pages or from title variants. The method computes the semantic relatedness between candidate pages and the context words using features derived from the following:

- Lexical overlap between article texts;
- Shared categories;
- Link structure, such as common incoming links.

The selected sense is the one whose page is most related to the surrounding context according to a similarity function.

This work demonstrates that Wikipedia can address the coverage limitations of traditional lexicons, especially for named entities, technical terms, and emerging concepts. It also shows that the hyperlink structure can be a substitute for manually constructed semantic relations.

#### 4.1.2. Link-Based Semantic Relatedness

Milne and Witten [8] introduced a widely used measure of semantic relatedness between Wikipedia concepts based on incoming links. Let Links(a) and Links(b) be the sets of pages that link to pages *a* and *b*, respectively. Relatedness is defined by an adaptation of the Normalized Google Distance (Equation (1)), where |W| is the total number of Wikipedia pages:

(1) $$\mathrm{rel}\left(a,b\right)=1-\frac{\log(\max(|\mathrm{Links}(a)|,|\mathrm{Links}(b)|))-\log(|\mathrm{Links}(a)\cap \mathrm{Links}(b)|)}{\log(|W|)-\log(\min(|\mathrm{Links}(a)|,|\mathrm{Links}(b)|))}$$

This measure captures the intuition that two pages are semantically related if many pages link to both. It is relatively robust to noise, does not require training, and can be precomputed or cached for efficiency. Many WSD and EL systems adopt this measure for the following:

- Ranking candidate entities by their relatedness to entities already selected in the context;
- Constructing coherence scores over sets of entities in global inference schemes.

#### 4.1.3. Explicit Semantic Analysis

Gabrilovich and Markovitch’s explicit semantic analysis (ESA) [4] represents texts as sparse, high-dimensional vectors over Wikipedia concepts. Each dimension corresponds to a Wikipedia article, and its weight reflects the association strength between the text and the article, typically computed using TF–IDF over an inverted index of Wikipedia.

ESA enables the following:

- Semantic similarity comparison between short texts and between words;
- Mapping of word contexts into concept distributions;
- Integration of encyclopedic knowledge into classification and retrieval tasks.

In WSD, ESA can be used by representing both the local context and each candidate sense page as vectors in the same space and selecting the sense whose vector is closest to the context vector. ESA also provides a generic mechanism for enriching document representations with Wikipedia-derived features that downstream models can exploit.

#### 4.1.4. Generalized Wikipedia-Based WSD

Li et al. proposed generalized WSD methods based on Wikipedia in [5,6]. Their approach treats Wikipedia as a universal knowledge base, integrating evidence from the following:

- Article content (bag-of-words and TF–IDF features);
- Category hierarchy;
- Hyperlinks and disambiguation pages.

The later two-stage disambiguation with Wikipedia (TSDW) model [6] decomposes the problem into the following steps:

1. **Coarse-grained disambiguation**, where simple features and prior probabilities derived from anchor statistics are used to select a subset of promising senses.
2. **Fine-grained reranking**, where richer semantic relatedness measures, including graph-based and content-based similarities, are used to rerank the candidates.

These works demonstrate how a collection of heterogeneous Wikipedia-derived signals can be systematically combined in a robust pipeline. They also illustrate the benefits of multi-stage architectures that separate efficient candidate pruning from more expensive semantic computations.

### 4.2. Unified WSD and Entity Linking with Wikipedia and BabelNet

#### 4.2.1. Babelfy and Unified Inventories

Babelfy [1] is a landmark system that unifies WSD and EL over BabelNet, a multilingual semantic network that integrates WordNet synsets, Wikipedia pages, and other resources. The system proceeds as follows:

1. For each content word or mention in the input text, it retrieves a set of candidate BabelNet synsets, some of which are backed by WordNet senses while others are backed by Wikipedia pages.
2. It constructs a semantic graph whose nodes are candidate synsets and whose edges represent semantic relations from BabelNet, such as lexical relations, encyclopedic relations, and gloss overlaps.
3. It performs a graph algorithm involving random walks with restart and densest subgraph identification to select a subset of nodes that are both well connected and relevant to the input text.

Because many BabelNet synsets correspond directly to Wikipedia pages or include them as part of a synset cluster, Babelfy can be viewed as a unified WSD/EL system grounded in Wikipedia entities. It does not distinguish sharply between named and common entities; all are nodes in a single graph.

Babelfy has been widely used as a reference system and as a component in pipelines that require concept annotation or semantic enrichment of text.

#### 4.2.2. Automatic Sense-Annotated Corpora: Train-O-Matic

Supervised WSD requires sense-annotated corpora, which are costly to create manually. Pasini and Navigli’s Train-O-Matic [9] addresses this by automatically generating large-scale sense-annotated corpora with minimal manual effort. The method works as follows:

- It uses BabelNet as the sense inventory, including Wikipedia-backed synsets.
- It identifies sentences where a target word is likely to be unambiguous, based on lexical and graph-based criteria.
- It assigns sense labels to occurrences where the context strongly favors a single candidate sense.

The resulting “silver-standard” corpora are orders of magnitude larger than manually annotated corpora and enable training of robust neural WSD models. Because many senses correspond to Wikipedia entities, these corpora implicitly encode mappings from raw text to Wikipedia-based senses.

#### 4.2.3. Multilingual Label Propagation: MuLaN

Barba et al.’s MuLaN [10] extends this idea to multilingual label propagation over the BabelNet graph. The key idea is that sense annotations in high-resource languages can be propagated to lower-resource languages by following inter-language links and graph edges. The process is as follows:

- Construct a multilingual graph whose nodes are senses (BabelNet synsets) and whose edges capture semantic relations and cross-lingual alignments.
- Use an initial set of labeled instances (e.g., from Train-O-Matic or existing sense-annotated corpora) as seeds.
- Propagate labels across the graph to annotate additional instances in multiple languages.

Because BabelNet is heavily grounded in Wikipedia, inter-language links between Wikipedia editions provide crucial alignment information. MuLaN thus illustrates how Wikipedia entities can function as multilingual pivots for WSD.

#### 4.2.4. Joint Word–Entity Embeddings from Wikipedia

Yamada et al. [25] proposed a model that learns embeddings for words and entities (Wikipedia pages) in a shared vector space. The model extends the skip-gram objective to incorporate the following:

- Word–word co-occurrences in text;
- Word–entity co-occurrences in contexts where entities are mentioned;
- Entity–word co-occurrences based on the words in their associated Wikipedia pages.

Sherkat and Milios [26] also studied vector embeddings of Wikipedia concepts and entities using graph and content information. These embeddings support the following:

- Ranking of candidate entities given a mention context;
- Computation of semantic relatedness between Wikipedia-based senses;
- Initialization of sense representations in neural WSD models.

Embedding methods provide dense, low-dimensional representations that can be readily integrated into neural architectures or used with similarity-based scoring functions.

#### 4.2.5. Wikipedia Graph-Based Entity Linking Pipelines

Beyond generic EL toolkits, Makris and Simos introduced systems dedicated to semantic annotation with Wikipedia entities. The work in [18] proposes techniques for text annotation where salient terms in a document are linked to coherent sets of Wikipedia entities. The method works as follows:

- It identifies candidate keyphrases or terms to annotate.
- It generates candidate entities using lexical and anchor-based matching.
- It selects a subset of entities that maximize semantic coherence, leveraging link-based relatedness measures reminiscent of [8] and concept-level similarities inspired by ESA [4].

Subsequent work extended this research into distributed and online settings. Makris et al. [20] proposed a distributed methodology for text semantic annotation based on community coherence, where communities of tightly related Wikipedia entities are discovered and used for annotation. Makris and Simos [19] introduced OTNEL, a distributed online deep-learning semantic annotation framework that updates entity representations incrementally as new documents arrive. Simos and Makris [21] presented a computationally efficient, context-free named entity disambiguation approach that exploits fuzzy logic and domain heuristics while retaining Wikipedia as the target knowledge base.

These systems illustrate how Wikipedia-based WSD and EL approaches can be engineered into scalable pipelines suitable for large corpora and real-world applications.

### 4.3. Neural and Contextualized Approaches

#### 4.3.1. Neural WSD: An Overview

The surveys by Wang et al. [3] and Bevilacqua et al. [2] documented the progression from knowledge-based and feature-engineered methods to neural architectures in WSD. Neural WSD systems typically have the following properties:

- They represent the context of a target word using distributed embeddings;
- They use classifiers, sequence models, or attention mechanisms to map contexts to sense distributions;
- They exploit large sense-annotated corpora, which are often automatically generated.

While many of these models were originally developed for WordNet-based senses, they can be readily adapted to Wikipedia-based inventories by changing the label space and using appropriate training data.

#### 4.3.2. Sense Embeddings and SensEmBERT

Scarlini et al. introduced SensEmBERT [13], which builds sense embeddings using contextualized representations from BERT. The procedure involves the following:

1. Collecting sense-annotated occurrences from corpora such as those produced by Train-O-Matic [9] and other resources.
2. Encoding each occurrence with a pretrained BERT model to obtain a context-sensitive vector for the target word.
3. Aggregating these vectors (e.g., by averaging) for each sense to produce a sense-specific embedding.

The resulting sense embeddings can be used for nearest-neighbor classification or retrofitting or as components in downstream models. When the sense inventory is BabelNet, many senses correspond directly or indirectly to Wikipedia entities, and thus these embeddings provide vector representations for Wikipedia-based senses.

In subsequent work, Scarlini et al. [12] showed that increasing the diversity and number of contexts per sense improved performance in all-round WSD. This observation has practical implications for constructing training corpora from Wikipedia; methods that extract many diverse contexts for each entity can produce more robust sense representations.

#### 4.3.3. Transformer-Based WSD Models

Transformer-based models have become dominant in many NLP tasks. Vandenbussche et al. [15] explored WSD with transformer encoders. Their approach treats WSD as a token classification problem:

- The input sentence is fed into a pretrained transformer (e.g., BERT or RoBERTa).
- A classification head predicts a probability distribution over senses for each target word.
- The model is fine-tuned on sense-annotated corpora.

When the label space consists of Wikipedia-based senses or BabelNet synsets, the same architecture applies. The main challenges are managing the large output space and addressing label sparsity. Techniques such as label smoothing, hierarchical classification, or tying parameters to sense embeddings can mitigate these issues.

Su et al. [14] proposed a multilingual WSD framework with unified sense representations. Their model has the following properties:

- It uses a multilingual transformer to encode contexts in multiple languages;
- It learns a shared embedding space for senses and contextual representations across languages;
- It exploits BabelNet as the sense inventory, relying on Wikipedia-derived cross- lingual links.

This framework is well suited to Wikipedia-based WSD, where multilingual alignment is intrinsic to the resource.

#### 4.3.4. Unsupervised Transformer-Based WSD

Ion et al. [11] examined unsupervised WSD using transformer attention patterns. Their method leverages the observation that transformer layers implicitly cluster contextual usages of words according to sense as follows:

- It extracts contextual representations for occurrences of ambiguous words;
- It clusters these representations to induce sense groups;
- It maps clusters to senses in an inventory, which may include Wikipedia-backed synsets, using similarity between cluster centroids and sense definitions or embeddings.

Such methods reduce dependence on sense-annotated training data and suggest that transformers encode latent sense distinctions that can be aligned with Wikipedia entities post hoc.

#### 4.3.5. Unsupervised and Knowledge-Based Enhancements

Rahman and Borah [27] proposed an unsupervised WSD approach that integrates distributional and knowledge-based cues. Although their work is not limited to Wikipedia, it can be adapted as follows:

- Using Wikipedia-derived semantic graphs instead of or in addition to WordNet;
- Exploiting link-based relatedness measures to guide clustering and selection.

Mizuki and Okazaki [28] investigated semantic specialization, where vector spaces are fine-tuned to better reflect the topology of a knowledge base. When Wikipedia entities are included in the knowledge graph (e.g., via BabelNet or Wikidata), specialization can increase the separation between similar but distinct senses and improve Wikipedia-based WSD.

#### 4.3.6. Neuro-Symbolic and Task-Oriented WSD

Zhang et al. [24] proposed a neuro-symbolic approach to sentiment analysis with dynamic WSD. Their system combines the following:

- Neural components that produce contextual embeddings;
- A symbolic reasoning module that selects senses and propagates sentiment information along a knowledge graph.

In settings where senses or entities correspond to Wikipedia pages, such frameworks can exploit encyclopedic information about entities, including sentiment-relevant cues in their descriptions, to disambiguate and interpret subjective content.

More generally, task-oriented WSD methods optimize sense disambiguation for a downstream objective (e.g., classification accuracy) rather than for intrinsic WSD metrics. When Wikipedia is the underlying KB, this implies designing models that select entities that are most informative for the downstream task.

### 4.4. Multilingual, Multimodal, and Low-Resource Settings

#### 4.4.1. Multilingual Label Propagation

MuLaN [10] demonstrated that multilingual label propagation over the BabelNet graph can yield high-quality sense-annotated corpora in many languages. The propagation process uses the following:

- Cross-lingual edges derived from inter-language links between Wikipedia editions;
- Semantic edges within each language derived from lexical and encyclopedic relations.

The ability to project sense labels from resource-rich languages (e.g., English) to languages with sparse annotations is particularly valuable in Wikipedia-based WSD, where coverage may vary significantly across languages.

#### 4.4.2. Multilingual Sense Inventories and Sense Bags

Patankar et al. [17] investigated the construction of a “Wiki sense bag” through multilingual WSD. They linked words in multiple languages to Wikipedia-based senses, thereby creating aligned sense inventories. This type of resource supports the following:

- Cross-lingual semantic similarity and translation selection;
- Multilingual information retrieval and question answering.

Su et al.’s unified multilingual sense representations [14] complement these efforts by providing a shared embedding space in which both word occurrences and senses (including Wikipedia entities) are embedded, facilitating cross-lingual disambiguation and transfer.

#### 4.4.3. WSD in Machine Translation

Raganato et al. [29] designed an evaluation benchmark to test the WSD capabilities of machine translation systems. The benchmark focuses on cases where sense distinctions yield different correct translations. Many of these cases involve named entities and domain-specific terms that are adequately modeled only when grounded in a knowledge base.

When sense labels are derived from BabelNet, Wikipedia entities indirectly influence which translations are considered correct. Evaluation results on such benchmarks highlight whether MT systems implicitly perform WSD and to what extent explicit Wikipedia-based WSD can improve translation quality.

#### 4.4.4. Multimodal and Visual WSD

Ogezi et al. [16] participated in SemEval-2023 Task 1 on multilingual visual WSD, in which the goal was to disambiguate words using both textual and visual context. The underlying sense inventories can include BabelNet synsets associated with images, many of which are aligned with Wikipedia pages.

Multimodal WSD opens future directions in which images associated with Wikipedia articles (e.g., infobox images or illustration figures) can be used as additional signals for disambiguation. For instance, visual features can help distinguish senses such as *apple* (fruit) and *Apple Inc.* (company) when the textual context is ambiguous.

#### 4.4.5. Low-Resource Languages

Although Wikipedia exists in many languages, their coverage is uneven. For low-resource languages, Wikipedia may contain only a small number of articles and a limited link structure. Multilingual approaches such as MuLaN [10] and that of Su et al. [14] partially mitigate this issue by projecting information from high-resource languages.

Further strategies include the following:

- Using parallel corpora and translation dictionaries to link low-resource language words to high-resource Wikipedia entities;
- Combining Wikipedia with local encyclopedias or specialized resources;
- Applying active learning or human-in-the-loop annotation to efficiently create gold data centered around Wikipedia entities.

#### 4.4.6. Cross-Lingual Entity Linking with Wikipedia as a Pivot

Beyond sense propagation, Wikipedia enables cross-lingual entity linking by providing aligned identifiers across language editions (via inter-language links) and shared cross-lingual cues such as titles, redirects, and anchor text. A common pattern is to (1) generate candidates from a high-resource Wikipedia edition (often English) using transliteration, translation, or multilingual dense retrieval and then (2) project or validate the prediction in the target language edition when available. This pivoting strategy is particularly effective when the target language has limited local link statistics but the entity exists in multiple editions.

#### 4.4.7. Aligning Wikipedia with Wikidata and Other Multilingual Resources

Many modern pipelines treat Wikipedia pages as surface-level descriptions and link them to a canonical, language-independent identifier such as a Wikidata Q-ID. This alignment supports multilingual transfer (a single identifier across languages), more principled NIL handling (explicitly representing entities that are missing from Wikipedia), and structured reasoning using Wikidata properties. In practice, Wikipedia–Wikidata alignment is also useful for normalizing aliases and resolving cross-edition inconsistencies that otherwise propagate to evaluation.

#### 4.4.8. Evaluation and Reporting Guidance for Multilingual and Low-Resource Settings

Multilingual Wikipedia-grounded evaluation can be brittle because failures may reflect inventory gaps rather than model errors. The reporting, when possible because of (1) per-language Wikipedia coverage for the target domains, (2) NIL rates and how NIL is operationalized, and (3) whether evaluation uses language-specific pages or language-agnostic identifiers (e.g., Wikidata Q-IDs), is particularly valuable. These diagnostics help separate representation errors from inventory limitations and make cross-paper comparisons more meaningful.

## 5. Evaluation

We summarize evaluation practice for Wikipedia-grounded WSD and EL, including widely used datasets and metrics.

### 5.1. Datasets, Benchmarks, and Evaluation

#### 5.1.1. Overview

Wikipedia-based WSD and EL systems are evaluated using a combination of traditional WSD benchmarks, automatically constructed corpora, and EL datasets with gold links to Wikipedia. The choice of evaluation data depends on the sense inventory and the intended application.

Table 4 summarizes representative datasets and benchmarks referenced by the surveyed methods.

#### 5.1.2. Traditional WSD Benchmarks

Classical WSD evaluation relies on datasets created for the Senseval and SemEval campaigns. These datasets use WordNet as the sense inventory and contain manually annotated sentences or documents. When a system operates on a different inventory (e.g., BabelNet or Wikipedia), evaluation requires a mapping between inventories.

Such mappings can be the following:

- Many-to-one, namely when multiple WordNet senses correspond to a single Wikipedia page or BabelNet synset;
- One-to-many, namely when a Wikipedia page covers several WordNet senses;
- Partial or noisy when no perfect correspondence exists.

These issues complicate direct comparisons between WordNet-based and Wikipedia-based systems. Some works restrict evaluation to those instances that can be mapped unambiguously; others treat mappings as approximate and accept some degree of error.

#### 5.1.3. Automatically Constructed Corpora

Train-O-Matic [9] and MuLaN [10] produce large sense-annotated corpora with BabelNet as the inventory. These corpora serve primarily as training data for supervised WSD models, but they can also be used for intrinsic evaluation, such as by holding out a portion as a validation set.

The main advantages of such corpora are their scale and coverage. The main disadvantages are noise and potential bias toward contexts where senses are easily disambiguated by the automatic heuristics. When Wikipedia entities are part of the inventory, the resulting corpora effectively encode Wikipedia-based WSD decisions.

#### 5.1.4. Entity Linking Benchmarks

From the EL perspective, standard corpora such as AIDA-CoNLL provide gold annotations linking mentions in a newswire to Wikipedia pages. Evaluation usually reports the precision, recall, and F_1_ score for correctly linked mentions with or without NIL detection.

Joint WSD/EL systems (e.g., Babelfy) are often evaluated on both WSD and EL benchmarks, demonstrating their ability to handle both named and common entities. When the focus is word sense disambiguation with Wikipedia entities, EL datasets provide valuable test cases for evaluating the disambiguation of named entities and proper nouns.

#### 5.1.5. Evaluation Metrics and Protocols

For WSD, the basic metric is accuracy, or the proportion of target instances for which the predicted sense matches the gold sense (after any mapping between inventories). Macro- or micro-averaging may be used depending on whether the goal is to weight all instances or all lemmas equally.

For EL, the mention-level precision, recall, and F_1_ score are standard, with additional metrics for NIL prediction and candidate ranking (e.g., mean reciprocal rank). Some work also considers document-level coherence or entity coverage.

When Wikipedia is used as a sense inventory, additional questions arise:

- Should redirects and page renames be treated as equivalent?
- How should disambiguation pages be handled when they do not correspond to a specific sense?
- How should predictions that refer to a page that has been merged or deleted since the dataset was created be treated?

Addressing these questions often involves fixing a particular Wikipedia snapshot and normalizing page identifiers via redirects.

#### 5.1.6. Challenges Specific to Wikipedia-Based Senses

Wikipedia-based WSD evaluation faces several specific challenges:

- **Dynamic inventory:** Wikipedia evolves over time, and thus gold annotations may refer to pages that no longer exist or whose content has changed significantly. Freezing a snapshot mitigates this but reduces alignment with current data.
- **Sense granularity:** The granularity of Wikipedia pages differs from WordNet and from fine-grained sense inventories. Some pages aggregate multiple senses (e.g., a page for a person that also represents the corresponding fictional character), whereas others split senses that might be indistinguishable for many applications.
- **Coverage biases:** Wikipedia coverage varies by language, domain, and topic. Certain entities and concepts are over-represented, while others are under-represented or absent.

Future evaluation frameworks may need to adopt graded measures, hierarchical labels (e.g., categories or Wikidata items), or task-specific notions of correctness.

## 6. Challenges and Future Directions

### 6.1. From Static Inventories to Dynamic Knowledge Ecosystems

Traditional WSD research has largely assumed static inventories such as WordNet, with well-defined sense sets and stable identifiers. Wikipedia-based WSD must operate in a dynamic environment, which has the following properties:

- Wikipedia pages are added, removed, renamed, and merged;
- New entities and concepts appear regularly;
- External knowledge bases (e.g., Wikidata) introduce new layers of structure and identifiers.

Future research should consider WSD as a continuous alignment problem between language usage and an evolving knowledge ecosystem. This may involve the following:

- Incrementally updating mappings between textual mentions and entities;
- Detecting and handling concept drift and changes in entity descriptions;
- Designing models that are robust to changes in the underlying knowledge base.

### 6.2. Integration with Large Language Models

Large language models (LLMs) encode substantial world knowledge implicitly in their parameters and can often resolve ambiguity without an explicit inventory. Probing work suggests that pretrained language models can store and recall factual associations, but this knowledge is latent, hard to audit, and can be inconsistent or outdated [30]. In contrast, Wikipedia-grounded WSD or EL yields an explicit decision: a mention is mapped to a concrete page identifier (or to NIL), enabling provenance, controlled updates, and stable evaluation.

#### 6.2.1. Implicit vs. Explicit Disambiguation

**Figure 3:**
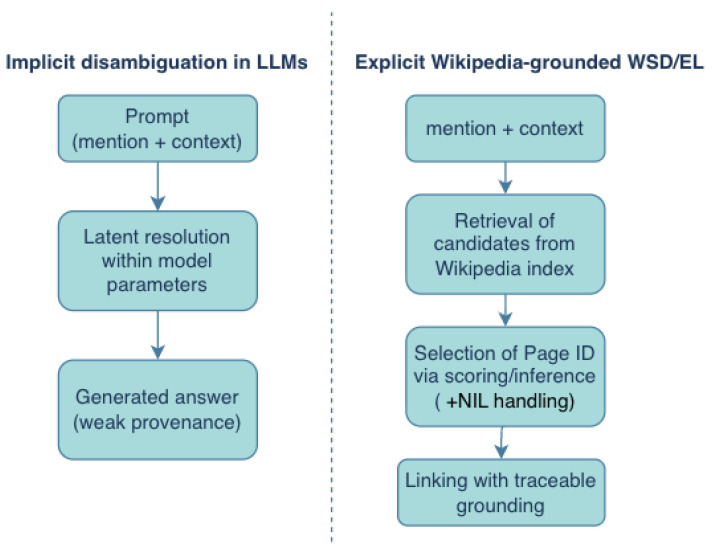
Implicit disambiguation in LLMs versus explicit Wikipedia-grounded WSD or EL. Explicit grounding provides a traceable identifier decision, which supports auditing, updating, and evaluation.

Figure 3 and Table 5 contrasts the two paradigms. While LLMs may perform *implicit* disambiguation as a side effect of next-token prediction, Wikipedia-grounded methods expose the intermediate linking decision and make the supporting evidence inspectable.

#### 6.2.2. Hybrid Systems: Retrieval and Grounding Around LLMs

Recent architectures increasingly combine parametric knowledge with retrieval over Wikipedia (or Wikipedia-derived indices), such as through retrieval-augmented pretraining and retrieval-augmented generation [31,32]. From the perspective of this survey, these systems can be interpreted as embedding a Wikipedia-style candidate retrieval step (and sometimes an identifier selection step) inside a generative pipeline. This makes explicit disambiguation components valuable even when the final output is conversational; the system can retrieve relevant pages, resolve ambiguous mentions to identifiers, and then condition generation on grounded evidence while retaining traceability.

#### 6.2.3. Opportunities at the Interface of WSD/EL and LLMs

Promising research directions include the following:

- **Retrieval-augmented disambiguation:** LLMs can be augmented with retrieval modules that fetch relevant Wikipedia pages at inference time, and disambiguation can be framed as selecting or reranking retrieved entities (see Figure 2).
- **Sense-aware prompting and constrained decoding:** Inputs can be enriched with candidate page summaries (or identifiers), and generation can be constrained to cite or condition on selected pages, strengthening auditability.
- **Joint training with explicit identifiers:** Models can be trained to predict Wikipedia page IDs as intermediate variables and use them as a bridge to downstream reasoning, aligning implicit representations with explicit inventories.

Overall, explicit Wikipedia grounding remains methodologically important as a controllable and evaluable layer, even when upstream components are LLMs that can perform implicit disambiguation.

### 6.3. Multilinguality and Low-Resource Conditions

While multilingual Wikipedia and BabelNet provide broad coverage, significant imbalances remain across languages. Future work on Wikipedia-based WSD in multilingual and low-resource conditions could explore the following:

- Improved graph-based label propagation that accounts for language-specific phenomena;
- Combination of Wikipedia signals with local resources, such as national encyclopedias or domain-specific lexicons;
- Better modeling of cross-lingual sense divergence, where concepts may not align one-to-one across languages.

Advances in multilingual transformers and cross-lingual representation learning will likely play a key role, but explicit Wikipedia-based alignment remains important for interpretability and integration with external knowledge.

### 6.4. Sense Granularity and Evaluation

Sense granularity remains a fundamental issue. Wikipedia pages differ from lexicographic senses in both coverage and granularity. For some applications, extremely fine-grained distinctions are unnecessary; for others, aggregated pages conflate distinct concepts.

Future evaluation methodologies may carry out the following:

- Adopt hierarchical or type-based scoring, where correctness is graded according to ontological proximity (e.g., via categories or Wikidata types);
- Provide application-specific evaluation settings that quantify the benefit of disambiguation at various granularities;
- Incorporate human judgments about the usefulness of distinctions for specific tasks.

These directions require closer interaction between WSD research and application domains.

### 6.5. Efficiency and Scalability

Scalable Wikipedia-based WSD is essential for large-scale semantic annotation and real-time applications. Challenges include the following:

- Efficient candidate generation and ranking in the face of large inventories;
- Distributed and streaming architectures, as illustrated by OTNEL [19] and community-coherence-based methods [20];
- Leveraging approximate nearest neighbor searches and compressed representations for large sense and entity embeddings.

Balancing accuracy and efficiency will remain a central practical concern, especially as neural models grow larger.

### 6.6. Answers to the Research Questions

We now summarize answers to the research questions stated in the Introduction:

- **RQ1 (Inventory design):** Wikipedia is operationalized as an inventory of page identifiers augmented by redirects, disambiguation pages, categories, and (in some systems) unified resources such as BabelNet. Inventory design mainly impacts granularity, coverage, and NIL behavior (Section 3 and Section 4).
- **RQ2 (Pipelines and inference):** Most systems decompose into candidate generation, local scoring, and optional global inference. Graph-based collective inference is most beneficial when multiple mentions are present and topical coherence can be enforced using a Wikipedia link structure (Figure 2; Section 4).
- **RQ3 (Representation learning):** Contextual encoders and ranking models improve local scoring by learning mention–page semantics from Wikipedia text and link supervision, while hybrid models retain graph constraints for global coherence (Section 4).
- **RQ4 (Multilinguality and robustness):** Wikipedia enables cross-lingual transfer via inter-language links and multilingual resources, while robustness depends on edition coverage and consistent identifier alignment. Reporting NIL rates and coverage diagnostics is critical in low-resource settings (Section 4).
- **RQ5 (LLM era):** LLMs can resolve ambiguity implicitly, but explicit Wikipedia grounding remains essential for auditability, updateability, and stable evaluation. Hybrid retrieval-augmented systems integrate these paradigms by retrieving and grounding based on Wikipedia evidence (Section 6).

## 7. Conclusions

The value of Wikipedia in the tasks of entity linking and word sense disambiguation has been established and well supported in numerous works. Its multilinguality and vast text data render it a particularly efficient sense inventory, without the traditional lexicographic source limitations. Early methods demonstrated the value of Wikipedia for entity linking through semantic linkages and concept models [4,5,6,7,8], with more recent approaches presenting unified approximations of WSD and EL on graph models [1,18,19,20,21], optimizing the sense annotation of auto-generated corpora [9,10].

There have been recent developments involving the use of neural and transformer models as far as contextualized representations of sense and multilingual disambiguation are concerned [11,12,13,14,15,27].

Sentiment analysis, domain-specific knowledge management, machine translation, and multimodal understanding applications [16,22,23,24] validate that accurate and interpretable entity linking with Wikipedia is particularly relevant for establishing semantic representations. Moving forward, the key challenges include integrating explicit grounding from Wikipedia into large language models, supporting multilingual and less-resourced languages, handling dynamic inventories, and designing evaluation protocols that correspond with application needs.

This survey sought to provide a comprehensive analysis of the design space of Wikipedia-based WSD and EL and emphasized connections throughout multiple traditions in a systematic manner, reviewing methods that treat Wikipedia entities as sense identifiers. We hope this will facilitate further progress at the intersection of lexical semantics, knowledge graphs, and large-scale neural models. 

## Figures and Tables

**Table 1:** Illustrative Wikipedia grounding for a common noun and a named entity.

Mention	Context	Candidate Targets (Wikipedia Pages)
Bank	“She deposited her paycheck at the bank.”	*Bank* (financial institution) vs. *River bank*
Jordan	“Jordan signed a contract with the Bulls in 1984.”	*Michael Jordan* vs. *Jordan* (country)/*Jordan River*

**Table 2:** Query template for identifying Wikipedia-grounded WSD and EL literature (illustrative; adapt to each library’s query syntax).

Component	Template
Wikipedia constraint	(Wikipedia OR BabelNet OR DBpedia)
Task constraint	(word sense disambiguation OR WSD OR entity linking OR named entity disambiguation OR concept linking)
Method hints (optional)	(graph OR collective OR neural OR contextual OR embedding)

**Table 3:** Taxonomy of Wikipedia-based WSD and entity linking approaches.

Category	Representative Works	Main Characteristics
Wikipedia-based relatedness and ESA	Mihalcea (2007) [7]; Milne and Witten (2008) [8]; Gabrilovich and Markovitch (2009) [4]	Use Wikipedia pages and links to compute concept-level similarity and disambiguate mentions via relatedness or vector space similarity.
Graph-based WSD or EL over Wikipedia or BabelNet	Li et al. (2011, 2013) [5,6]; Moro et al. (2014) [1]; Makris and Simos (2014) [18] Makris and Simos (2020) [19]; Simos and Makris (2022) [21]	Model candidate senses as nodes in a graph (often including Wikipedia entities) and perform global inference or label propagation to enforce coherence.
Automatic sense-annotated corpora and label propagation	Pasini and Navigli (2020) [9]; Barba et al. (2020) [10]; Makris et al. (2020) [20]	Generate large-scale sense annotations by exploiting unambiguous anchors and graph-based propagation, usually on BabelNet or Wikipedia graphs.
Neural and contextualized models	Scarlini et al. (2020) [12,13]; Vandenbussche et al. (2021) [15]; Su et al. (2022) [14]; Ion et al. (2025) [11]	Fine-tune neural encoders or build sense embeddings over automatically labeled corpora, often grounding senses in Wikipedia-backed inventories.

**Table 4:** Representative datasets and benchmarks used in Wikipedia-based WSD and entity linking.

Dataset or Benchmark	Task	Sense Inventory or KB	Notes
Traditional WSD benchmarks (Senseval or SemEval)	All-words WSD	WordNet; sometimes mapped to BabelNet or Wikipedia	Standard WSD datasets, often remapped when systems use Wikipedia-based inventories [2,3].
Train-O-Matic corpora [9]	All-words WSD	BabelNet (WordNet + Wikipedia)	Automatically generated sense-annotated corpora for multiple languages; widely used to train neural WSD models
MuLaN corpora [10]	Multilingual WSD	BabelNet or Wikipedia	Label-propagated multilingual sense annotations aligned across languages via Wikipedia entities
Raganato MT WSD benchmark [29]	WSD in MT	BabelNet	Tests sensitivity of MT systems to sense distinctions that significantly impact translation quality
Visual WSD (SemEval-2023 Task 1) [16]	Multilingual visual WSD	BabelNet or Wikipedia	Combines textual and visual context; many senses correspond to Wikipedia entities
Wikipedia-based EL corpora (e.g., AIDA-CoNLL)	Entity linking	Wikipedia	Mentions in news or web text linked to Wikipedia pages; often used for evaluating joint WSD/EL systems such as Babelfy [1].

**Table 5:** Methodological contrast between implicit LLM disambiguation and explicit Wikipedia-grounded WSD or EL.

Dimension	Implicit (LLM-Internal)	Explicit (Wikipedia-Grounded)
Provenance	Often unavailable or post hoc	Page-level identifiers and retrievable evidence
Updateability	Requires retraining or model editing	Update index and inventory snapshot independently
Evaluation	Hard to isolate the linking decision	Directly measurable linking or WSD accuracy and NIL behavior
Control	Limited (prompting and decoding)	Constrained candidate sets, explicit NIL policies
Failure modes	Hallucinations, unstable attribution	Inventory gaps, candidate generation errors

## Data Availability

No new data were created or analyzed in this study.

## Abbreviations

The following abbreviations are used in this manuscript:

NED	Named entity disambiguation
EL	Entity linking
WSD	Word sense disambiguation
